# Photoactivatable Large Stokes Shift Fluorophores for Multicolor Nanoscopy

**DOI:** 10.1021/jacs.2c12567

**Published:** 2023-01-10

**Authors:** Ilya Likhotkin, Richard Lincoln, Mariano L. Bossi, Alexey N. Butkevich, Stefan W. Hell

**Affiliations:** † Department of Optical Nanoscopy, Max Planck Institute for Medical Research, Jahnstrasse 29, 69120 Heidelberg, Germany; ‡ Department of NanoBiophotonics, Max Planck Institute for Multidisciplinary Sciences, Am Fassberg 11, 37077 Göttingen, Germany

## Abstract

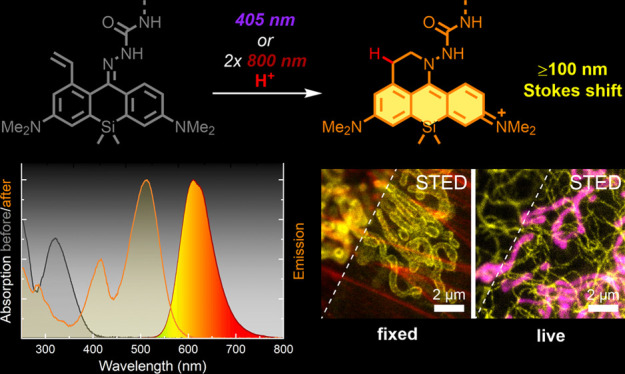

We designed caging-group-free photoactivatable live-cell permeant dyes with red fluorescence emission and ∼100 nm Stokes shifts based on a 1-vinyl-10-silaxanthone imine core structure. The proposed fluorophores undergo byproduct-free one- and two-photon activation, are suitable for multicolor fluorescence microscopy in fixed and living cells, and are compatible with super-resolution techniques such as STED (stimulated emission depletion) and PALM (photoactivated localization microscopy). Use of photoactivatable labels for strain-promoted tetrazine ligation and self-labeling protein tags (HaloTag, SNAP-tag), and duplexing of an imaging channel with another large Stokes shift dye have been demonstrated.

Fluorescent dyes with large Stokes shifts are valuable tools in multicolor fluorescence microscopy, since they expand color multiplexing^1^ and allow shifting the detection window beyond the range of cell autofluorescence^2^ (<580 nm). These fluorophores typically have ≥80 nm difference between the excitation and emission band maxima for the lowest energy electronic transition (or ≥2000–4000 cm^–1^, depending on the spectral range). Red- and near-infrared (NIR) emitting fluorophores are particularly interesting,^3^ because they reduce phototoxicity and enable deeper optical penetration into the sample. Fluorophores with very large Stokes shifts demonstrate zero overlap between absorption and emission spectra, resulting in no self-quenching by reabsorption, and enable the quantitative interpretation of fluorescence signal irrespective of the label concentration.^4^

The growing usage of super-resolution light microscopy (nanoscopy) in routine biological imaging increases the demand for small-molecule photoactivatable or photoswitchable fluorescent probes.^5^ Unfortunately, there have been but singular reported examples of photoactivatable^6^ and photoconvertible^7^ large Stokes shift fluorophores, with none of them being suitable for tagging arbitrary proteins in living cells.

Light-induced protonation was previously reported for a photoactivatable PA-SiR dye,^8^ which is converted into an electrophilic Si-pyronine fluorophore with limited chemical stability (Figure 1a). We have recently proposed a general synthetic strategy toward compact and biocompatible photoactivatable 1-vinyl-3,6-diaminoxanthone derivatives (PaX dyes^9^). Upon irradiation with UV or visible light, these compounds undergo rapid and clean intramolecular 6*-endo-trig* cyclization into the corresponding xanthylium fluorophores (Figure 1b) without generation of reactive electrophilic or radical intermediates and avoiding the need for photolabile protecting (caging) groups. Combining this idea with our earlier design of live cell-compatible large Stokes shift fluorescent probes,^10^ based on the pioneering works by Klán^11^ and Burgess,^12^ we have developed and report herein caging-group-free photoactivatable fluorophores with Stokes shifts ≥100 nm.

**Figure 1:**
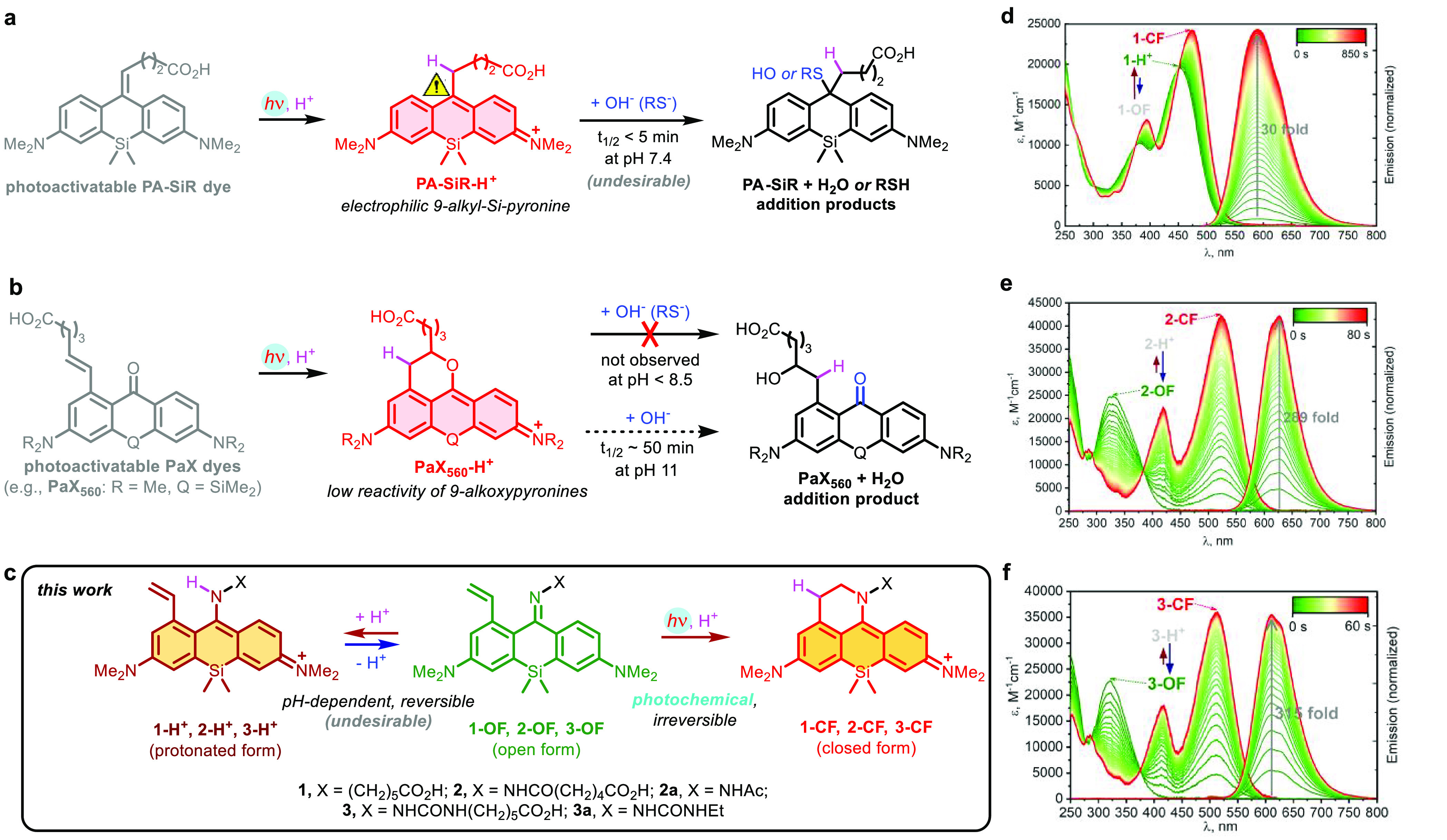
Photoactivatable large Stokes shift fluorescent dyes based around Si-pyronine core structure. (a) Photoactivatable PA-SiR dye^8^ generating an electrophilic 9-alkyl-Si-pyronine fluorophore with ∼20 nm Stokes shift. (b) Photoactivatable 1-vinylxanthone (PaX) dyes are stable toward nucleophiles under neutral conditions.^9^ (c) Reversible protonation of 1-vinyl-10-silaxanthone imines **1-OF**–**3-OF** vs an irreversible photochemical ring closure. (d–f) Changes in absorption and fluorescence emission spectra of **1-OF** (d), **2-OF** (e), and **3-OF** (f) upon UV irradiation (405 nm, d; 365 nm, e,f).

In our initial attempt, an imino analogue **1** of the PaX_560_ core^9^ was prepared (Figure 1b). Even though this dye demonstrated sufficient fluorescence increase upon activation with 405 nm (or 455 nm) wavelength LED light in aqueous media (30-fold in phosphate buffer at pH 7.0, Figure 1d) and the staining with secondary antibodies was specific, the fluorescence intensity before photoactivation in samples immunostained against tubulin was found unacceptably high (Figure S1), resulting in only 2-fold emission enhancement upon UV irradiation. We hypothesized that switching from benzophenone imine to benzophenone oxime or hydrazone analogues should lower the contribution of the unwanted protonated state (e.g., **1-H**^**+**^) for the unactivated **OF** form of the dye. Following a brief *N*-substituent screening, an acyl hydrazone **2** and a semicarbazone **3** were identified as maintaining sufficiently large Stokes shift of the activated forms **2-CF**, **3-CF** while showing a clear preference for imine forms **2-OF**, **3-OF** before photoactivation (Figure 1e,f) at physiologically relevant pH values. The dyes **2** and **3** demonstrated virtually indiscernible absorption and emission spectral profiles with a one unit p*K*_a_ difference (Figure S2a,b and Table 1), and underwent complete and byproduct-free photoactivation (Figure S3a,b). On studying the pH behavior of the model compounds, we noticed that the fluorescent closed form **2a-CF** was losing its long-Stokes emission above pH > 7 (possibly due to deprotonation of the hydrazone NH; Figure S2c), while the photoactivated semicarbazone derivative **3a-CF** remained pH-insensitive across the entire pH 3–10 range (Figure S2d). We further investigated the photostability of the activated form of these model dyes (Figure S4) in neutral buffers and found the stability of the fluorophores lying within the range of commercial large Stokes shift labels *DyLight 515-LS* and *abberior LIVE 460L*, with **3a-CF** being more photostable than **2a-CF**.

**Table 1:** Properties of Photoactivatable Large Stokes Shift Dyes **1**–**3** before and after Activation

before photoactivation				after photoactivation					
dye	absorption λ_max_, nm (ε, M^–1^ cm^–1^)	p*K*_a_	Φ_Act_^a^	dye	absorption λ_max_, nm (ε, M^–1^ cm^–1^)	emission λ_max_, nm (Φ_fl_^b^)	p*K*_a_	Stokes shift, nm (cm^–1^)	fluorescence lifetime τ, ns
**1-H**^**+**^	457 (20450)	>10.5	0.001^c^	**1-CF**	470 (23360)	590 (0.28)	n.d.	120 (4328)	2.61
**2-OF**	329 (24800)	4.0	0.023^d^	**2-CF**	523 (43150)	627 (0.45)	7.5	104 (3171)	2.87
**3-OF**	321 (22800)	5.3	0.036^d^	**3-CF**	513 (36050)	611 (0.45)	>10.5	98 (3126)	3.00

a Photoactivation quantum yield.

b Fluorescence quantum yield.

c Irradiated at 405 nm.

d Irradiated at 365 nm.

We initiated our imaging studies by immunostaining the tubulin filaments in fixed COS-7 fibroblast-like cells with secondary antibodies labeled with reactive esters **2-NHS** or **3-NHS** (Figure 2a). Both labels could be activated in one-photon or two-photon mode with a 405 or 800 nm activation laser, respectively (Figure S5, S6), and were found to be compatible with high peak intensity (∼60 MW/cm^2^) of 775 nm pulsed STED light, allowing to resolve the individual filaments with subdiffraction resolution (Figure 2b,c and Figure S7). However, the samples labeled with **2-NHS** demonstrated non-negligible initial fluorescence (24% for **2-NHS** vs 5% for **3-NHS**) and required prebleaching of the label with 485 or 518 nm laser to approach zero background intensity levels before activation. For this reason, the dye **3** was preferred for single-molecule localization imaging. With the probe **3-Halo**, it was possible to resolve the circular shape of individual nuclear pores (outer diameter 107 nm^13^) in fixed U2OS cells stably expressing one of the nuclear pore complex proteins (Nup96) as a fusion with the HaloTag protein (a self-labeling modification of *Rhodococcus rhodochrous* chloroalkane dehalogenase DhaA^14^) using photoactivated localization microscopy (PALM; Figure S8, S9).

**Figure 2:**
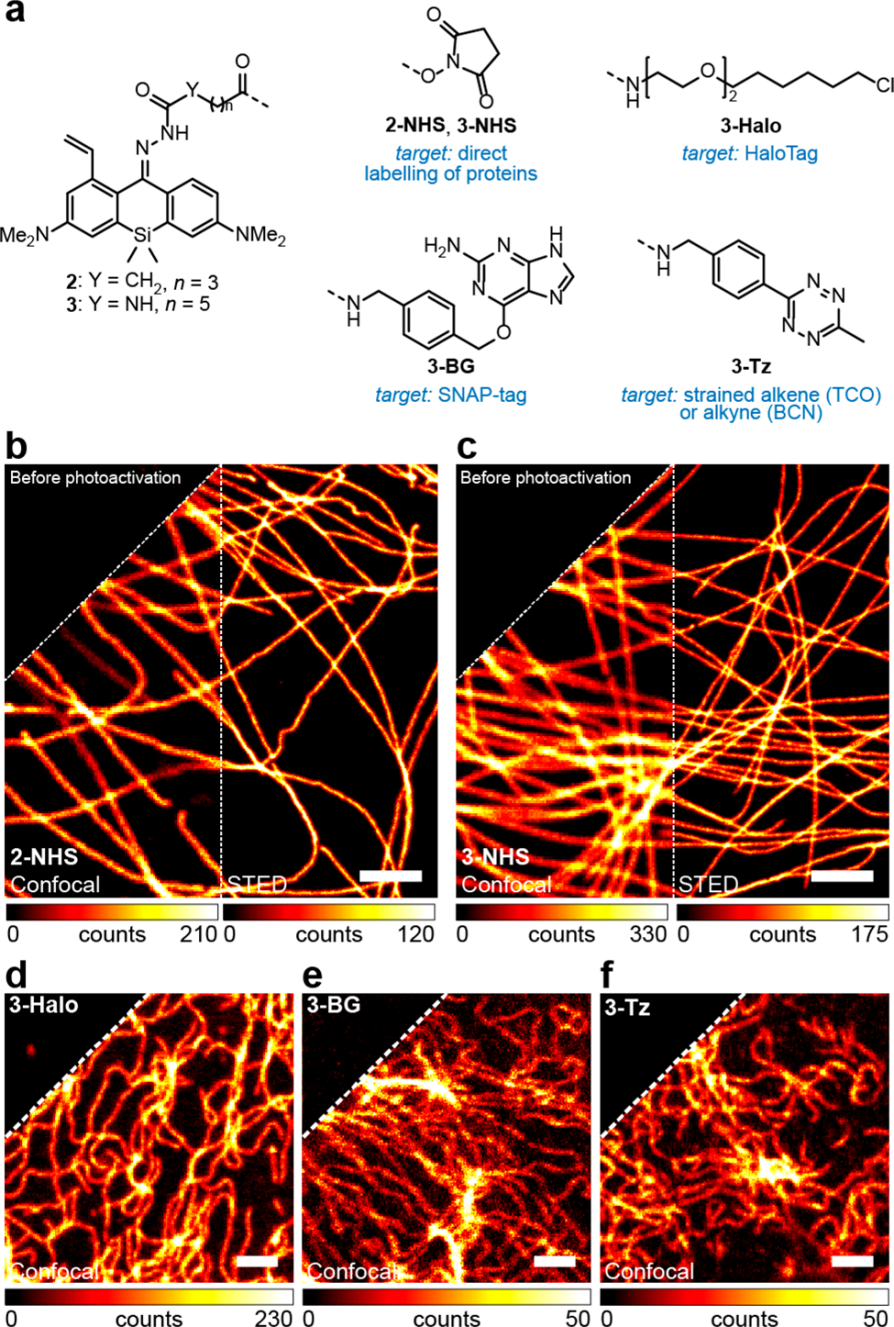
(a) Photoactivatable large Stokes shift fluorescent labels for fixed- (b,c) and live-cell (d–f) fluorescence imaging. (b,c) Confocal and STED images of tubulin filaments in fixed COS-7 cells labeled by indirect immunofluorescence with secondary antibodies tagged with **2-NHS** (b) or **3-NHS** (c). (d) Confocal image of U2OS cells stably expressing vimentin-HaloTag fusion protein labeled with **3-Halo** (200 nM). (e) Confocal image of U2OS cells stably expressing vimentin-SNAP-tag fusion protein labeled with **3-BG** (1 μM). (f) Confocal image of U2OS cells stably expressing vimentin-HaloTag fusion protein labeled with HTL-BCN (10 μM) followed by **3-Tz** (1 μM). In the upper left corner of (b–f), the same images before photoactivation are shown. Scale bars: 2 μm.

To validate the selectivity of intracellular targeting with the ligands derived from photoactivatable large Stokes shift dye **3** in living cells, several labeling strategies have been explored. First, a vimentin-HaloTag fusion protein stably expressed in U2OS cells, genetically engineered using CRISPR-Cas9 technology (U2OS-Vim-Halo cells^15^), was labeled directly using the **3-Halo** ligand. The indirect labeling was performed by pretreating the cells with excess bicyclo[6.1.0]non-4-yne (BCN) HaloTag ligand (HTL-BCN) followed by treatment with the tetrazine derivative **3-Tz**. Alternatively, living U2OS cells with stable expression of vimentin fused with SNAP-tag protein^15^ (an engineered human *O*^6^-alkylguanine-DNA alkyltransferase AGT^16^) were stained with *O*^6^-benzylguanine ligand **3-BG**. As illustrated by the Figure 2d–f, in all cases the bright and high-contrast selective staining of vimentin filaments has been achieved, with very little fluorescent background preceding the activation of the dye and no visible off-targeting in the activated samples. Besides these universally applicable self-labeling protein tags, selective targeting of **3** to lysosomes (Figure S10) was confirmed for a noncovalent Pepstatin A ligand (a potent inhibitor of the ubiquitous lysosomal aspartic protease cathepsin D, binding to its active form^17^) by colocalization with the commercial *SiR-lysosome* probe.^18^

Relatively low sensitivity of the photoactivatable dyes **2** and **3** to visible light (>450 nm) permits channel duplexing with another large Stokes shift fluorophore sharing the same excitation wavelength and detection window. In our example, this scheme was realized in an immunostained sample by sequential imaging of the microtubules labeled with a coumarin dye *DyLight 515-LS* (excitation λ_max_ 515 nm, emission λ_max_ 650 nm) followed by photobleaching with the 485 nm excitation laser (Figure 3a,b). Photoactivation of **3**-labeled secondary antibodies used in indirect immunofluorescence labeling of TOMM20 protein was then achieved with 405 nm light (Figure 3c) and followed by confocal and STED imaging of mitochondria using identical instrument detection settings. This sequence provided a pseudo-two-color image for two large-Stokes fluorophores with overlapping absorption and emission spectra (Figure 3d–g).

**Figure 3:**
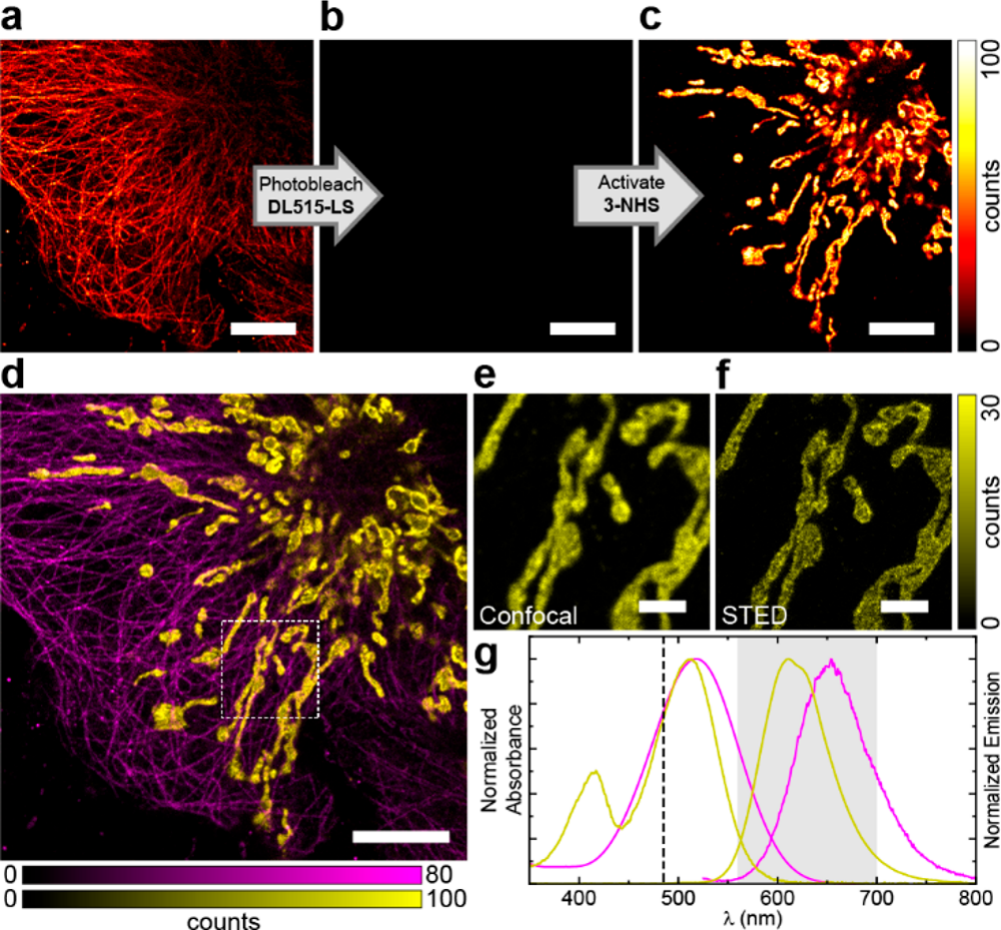
Channel duplexing with photoactivatable large Stokes shift dye **3**. (a–c) Confocal imaging of fixed COS-7 cells with microtubules labeled by indirect immunofluorescence with *DyLight 515-LS* NHS ester (ThermoFisher) and mitochondria labeled with **3-NHS** before (a) and after (b) photobleaching of *DyLight 515-LS* and after (c) photoactivation of **3** by a 405 nm laser. (d) Combined pseudo-two-color image showing microtubules (magenta) and mitochondria (yellow) obtained by sequential imaging as shown in (a–c). (e,f) Confocal (e) and STED (f) images of mitochondria in the magnified region marked in (d). (g) Absorption and emission spectra of *DyLight 515-LS* (magenta) and **3** (yellow), the excitation laser line (485 nm, dashed line), and the detection window (560–700 nm, gray); STED wavelength of 775 nm. Scale bars: 10 μm (a–d), 2 μm (e–f).

Finally, we demonstrated the use of photoactivatable large Stokes shift labels in four-color confocal imaging of fixed and living mammalian cells (Figure 4). In glutaraldehyde-fixed COS-7 cells, TOMM20 protein in mitochondria and vimentin in intermediate filaments were labeled by means of secondary antibodies tagged with **3-NHS** and the NHS ester of a highly photostable anionic rhodamine dye *abberior STAR 512*,^19^ respectively, with the actin cytoskeleton and nuclei counterstained with *SiR-actin*(20) and the DNA binder 4′,6-diamidino-2-phenylindole (DAPI). Two-color imaging could be performed first, followed by photoactivation of **3** with 405 nm laser line and recording the data for **3** and DAPI (Figure S11a). In living U2OS-Vim-Halo cells, photoactivatable HaloTag ligand **3-Halo** was employed along with live-cell compatible pentamethine cyanine mitochondrial label *MitoTracker Deep Red FM*,^21^ a cell-permeant nuclear counterstain Hoechst 33342, and the noncovalent small molecule *abberior LIVE 510-tubulin* probe (a cabazitaxel-tagged rhodamine dye^22^). Since the latter is less photostable as compared to *STAR 512*, partial bleaching of the labeled vimentin filaments was observed in the second acquisition (Figure S11b). In both cases, complete color separation in all four channels required the use of spectral unmixing.^23^

**Figure 4:**
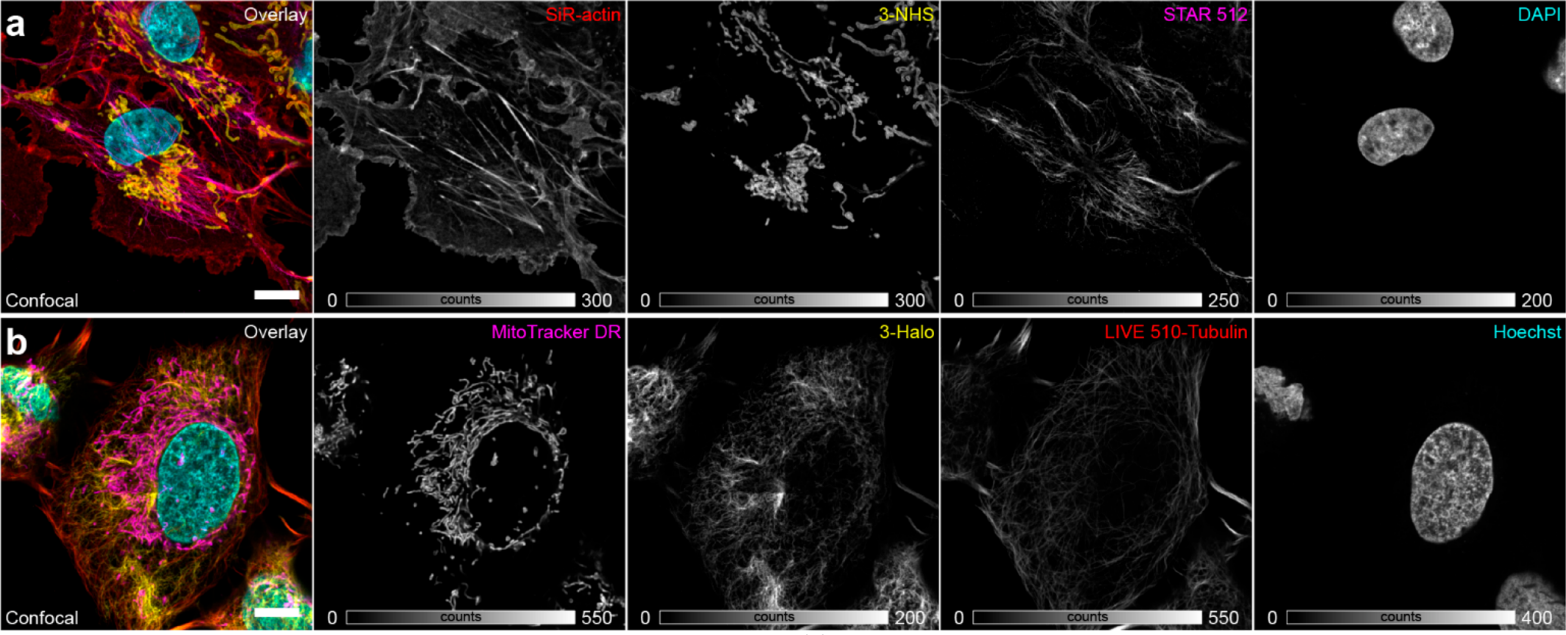
Multicolor imaging with photoactivatable large Stokes shift labels. (a) Confocal imaging of fixed COS-7 cells labeled by indirect immunofluorescence for mitochondria (**3-NHS**) and vimentin (*abberior STAR 512* NHS ester), and with small-molecule fluorescent probes *SiR-actin* (for F-actin) and DAPI (for nuclear DNA). (b) Confocal imaging of living U2OS cells stably expressing a vimentin-HaloTag construct labeled with **3-Halo** and live cell-compatible probes *MitoTracker Deep Red FM* (specific for mitochondria), *abberior LIVE 510-tubulin* (microtubules), and Hoechst 33342 (DNA). Spectral unmixing of individual channels was performed according to ref (23). Scale bars: 10 μm.

In conclusion, we introduced live-cell membrane-permeant photoactivatable fluorescent dyes with large Stokes shifts (∼100 nm) based on a caging-group-free activation mechanism unique for PaX fluorophores.^9^ Similarly to these small Stokes shift fluorophores (<40 nm), the best-performing dye **3** was found compatible with both STED (at 775 nm) and PALM fluorescence nanoscopy, each imposing distinct demands on the fluorescent label. Photoactivatable ligands derived from dye **3** permit up to four-color microscopy in fixed and live samples and form two-color combinations with commercial large Stokes shift probes based on a photobleaching–photoactivation sequence, which can be trivially realized with commercial fluorescence microscopes. We also anticipate the application of our photoactivatable large Stokes shift labels in multicolor single-molecule tracking of labeled biomolecules, further expanding the toolkit available for studying biochemical mechanisms.

## Supporting Information Available

Additional experimental details, materials, methods, and characterization data for all new compounds (PDF)
